# High Risk versus Proportional Benefit: Modelling Equitable Strategies in Cardiovascular Prevention

**DOI:** 10.1371/journal.pone.0140793

**Published:** 2015-11-03

**Authors:** Ivanny Marchant, Jean-Pierre Boissel, Patrice Nony, François Gueyffier

**Affiliations:** 1 Escuela de Medicina, Universidad de Valparaíso, Valparaíso, Chile; 2 NovaDiscovery, Biomodelling department, Lyon, France; 3 CNRS, UMR 5558, Laboratoire de Biométrie et Biologie Evolutive, Service de Pharmacologie Clinique et Essais Thérapeutiques, Lyon, France; 4 INSERM, CIC 201, EPICIME, Lyon, France; 5 Hop L Pradel, CHU Lyon, Lyon, France; INRCA, ITALY

## Abstract

**Objective:**

To examine the performances of an alternative strategy to decide initiating BP-lowering drugs called Proportional Benefit (PB). It selects candidates addressing the inequity induced by the high-risk approach since it distributes the gains proportionally to the burden of disease by genders and ages.

**Study Design and Setting:**

Mild hypertensives from a Realistic Virtual Population by genders and 10-year age classes (range 35–64 years) received simulated treatment over 10 years according to the PB strategy or the 2007 ESH/ESC guidelines (ESH/ESC). Primary outcomes were the relative life-year gain (life-years gained-to-years of potential life lost ratio) and the number needed to treat to gain a life-year. A sensitivity analysis was performed to assess the impact of changes introduced by the ESH/ESC guidelines appeared in 2013 on these outcomes.

**Results:**

The 2007 ESH/ESC relative life-year gains by ages were 2%; 10%; 14% in men, and 0%; 2%; 11% in women, this gradient being abolished by the PB (relative gain in all categories = 10%), while preserving the same overall gain in life-years. The redistribution of benefits improved the profile of residual events in younger individuals compared to the 2007 ESH/ESC guidelines. The PB strategy was more efficient (NNT = 131) than the 2013 ESH/ESC guidelines, whatever the level of evidence of the scenario adopted (NNT = 139 and NNT = 179 with the evidence-based scenario and the opinion-based scenario, respectively), although the 2007 ESH/ESC guidelines remained the most efficient strategy (NNT = 114).

**Conclusion:**

The Proportional Benefit strategy provides the first response ever proposed against the inequity of resource use when treating highest risk people. It occupies an intermediate position with regards to the efficiency expected from the application of historical and current ESH/ESC hypertension guidelines. Our approach allows adapting recommendations to the risk and resources of a particular country.

## Introduction

The introduction of the absolute CVD risk as a cornerstone of hypertension management[1] has increased the efficiency of cardiovascular prevention in comparison to the classical recommended BP level alone to identify the target population to BP-lowering drugs[2]. However, the high absolute risk strategy has been criticized by the European Joint Prevention guidelines since the 2003 version[3], mainly because it induces the concentration of health resources on the oldest men due to their highest risk, while young patients at high relative risk, especially women, may often not reach treatment thresholds. Elderly people may be overexposed to treatments, would the absolute risk rule be strictly applied. Yet, as there is no natural risk threshold over which all the benefit from treatment would be observed, the choice has relied on cost-effectiveness principles adopting a unique risk threshold disregarding the age or gender of hypertensive individuals. If treatment effect, expressed as relative risk, is the same whatever the risk level, individuals at the highest risk gain most from risk factor management. However, most deaths in a population occur in low-risk individuals because they are more numerous than high-risk individuals[4]. Moreover, these deaths have heavier consequences in terms of years of life lost. Younger persons at low absolute but high relative risk may well benefit from risk factors control in their future life[5], delaying the occurrence of cardiovascular events. To address the issue of low absolute but high relative risk, the 2007 European Joint Guidelines[6] encourage comparing the individual risk to the risk of peers, i.e. individuals of the same gender and age with ideal levels of risk factors, to decide initiating lifestyle measures to reduce the CVD incidence in low-risk individuals. Revisiting this problem, the latest 2012 version of these Guidelines[1] introduce the risk age approach. The cardiovascular risk age of a person with several cardiovascular risk factors is the age of somebody with the same level of risk but with ideal levels of risk factors. A 40-year old individual may have a risk age of 60 years; illustrating the likely reduction in life expectancy these individuals will be exposed to if preventive measures are not adopted. Nevertheless, no threshold of relative risk has been established nor the question of when drugs treatment would be advisable in this subgroup of individuals has been addressed. The latest guidelines for hypertension management of 2013 stress the importance of the level of evidence when selecting the patients that should be treated with BP-lowering drugs[7]. Unfortunately, the scarcity of evidence does not mean that treatment is indeed inefficacious in some specific population subgroups. It remains difficult to justify leaving untreated those individuals for whom the evidence on treatment benefits is weak.

Attempts have been made to provide arguments for a more rational decision process, and lifetime CVD risk prediction models[8] have met increasing interest in providing justification for treating younger individuals at low short-term risk but high lifetime risk. From a less enthusiastic position regarding the irreconcilable dimensions of the potential loss of life due to untreated CVD risk, i.e. greater loss of life expectancy among the younger versus greater number of lives lost due to age-dependent risk increase, many economists hold the view that future health gains should be discounted[9–12], arguing that people give decreasing value to expected benefits in their future life compared to current life[13]. Discounting life expectancies by time preference rates would thus result in higher relative value for the remaining life of older individuals compared to younger individuals if time preference-discounting, case fatality rates and competing risks are considered to estimate the impact of patient age at onset of treatment on the expected benefits of cardiovascular preventive treatments[14].

But how these single, societal or economical criteria could be validated? The fundamental question of which principles should govern the definition of the treatment target population remains unsolved. We propose here a method for the implementation of the peers’ risk approach based on a proportional benefit, derived from solid data largely available with an appropriate reliability: the incidence of cardiovascular deaths within the different categories of gender and age in a population and the proportion of deaths that could be avoided from the overall resources available for prevention. The resulting treatment strategy offers the same Proportional Benefit, or the part of the predicted loss of life that would be prevented by treating the individuals eligible, independently from their age or gender. We assessed the efficacy and efficiency of the Proportional Benefit strategy against the 2007 ESH/ESC recommendation[5] at the scale of a country population, represented by the French Realistic Virtual Population (RVP)[15].

## Methods

According to the 2007 ESH/ESC recommendation, all the individuals having a BP level greater or equal to 160/100 mm Hg have to be prescribed BP-lowering drugs independently from their CVD risk and conversely, individuals with a BP level < 130/85 mm Hg have no indication of antihypertensive drugs. All these individuals were thus excluded from the analyses and we focused only on the individuals for whom the prescription of BP-lowering drugs was based on the BP level combined with the CVD risk criterion, henceforth called individuals “potentially eligible”: individuals with BP levels in the high normal range and grade 1 hypertension. For the seek of demonstration, we established a reference “conservative” prescription scenario based on the 2007 ESH/ESC guidelines where all drug indications that were recommended once lifestyle measures had failed were considered as definite drug indications. Thus, in the reference prescription scenario, individuals with high-normal BP at high risk as well as those with grade 1 hypertension and moderate to high CVD risk were eligible to drugs treatment, disregarding the age.

The 2013 guideline version introduced modifications to the 2007 guidelines mainly based on the level of trial evidence [7]. The 2013 guidelines: 1) exclude high-normal BP individuals from drugs prescription, 2) consider grade 1 hypertension and low risk individuals as eligible to treatment (although it cannot be strongly recommended since evidence is weak) and 3) recommend that, in elderly patients, drug treatment should be initiated only when SBP is ≥ 160 mm Hg, although it may be considered when SBP is in the 140–159 mm Hg range. We established two new prescription scenarios assuming different interpretations of the 2013 guidelines: 1) an evidence-based scenario: excluding grade 1 low risk and grade 1 elderly patients from treatment, and 2) an opinion-based one: including grade 1 low risk and grade 1 elderly patients (Table 1).

**Table 1:** Prescription scenarios according to ESH/ESC successive guidelines and the Proportional Benefit strategy.

**2007 ESH/ESC guidelines**			**Proportional Benefit strategy**			
	Blood Pressure (mm Hg)		CVD risk >	Age class	Blood Pressure (mm Hg)	
	High normal	Grade 1 HT	percentile		High normal	Grade 1 HT
	SBP 130–139	SBP 140–159	adjusted to		SBP 130–139	SBP 140–159
CVD risk	or DBP 85–89	or DBP 90–99	the peers' risk		or DBP 85–89	or DBP 90–99
Low						
< 1%			65	35–44	Drugs	Drugs
Moderate		Drugs				
≥ 1% & < 5%			64	45–54	Drugs	Drugs
Increased	Drugs	Drugs				
≥ 5%						
Elderly patients	Drugs	Drugs	61	55–64	Drugs	Drugs
> 60 years						
**2013 ESH/ESC guidelines opinion-based**				**2013 ESH/ESC guidelines evidence-based**		
	Blood Pressure (mm Hg)				Blood Pressure (mm Hg)	
	High normal	Grade 1 HT			High normal	Grade 1 HT
	SBP 130–139	SBP 140–159			SBP 130–139	SBP 140–159
CVD risk	or DBP 85–89	or DBP 90–99		CVD risk	or DBP 85–89	or DBP 90–99
Low		Drugs		Low		
< 1%				< 1%		
Moderate		Drugs		Moderate		Drugs
≥ 1% & < 5%				≥ 1% & < 5%		
Increased		Drugs		Increased		Drugs
≥ 5%				≥ 5%		
Elderly patients		Drugs		Elderly patients		
> 60 years				> 60 years		

We designed the Proportional Benefit strategy as an alternative approach to select the individuals eligible to BP-lowering drugs treatment while attaining at least the same overall benefit in terms of life-years gained as with the 2007 European guideline application. This benefit was distributed proportionally, i.e. with the same ratio of number of fatal CVD events to prevent over the expected number of fatal CVD incident events across the different categories of individuals. The desired number of events prevented was used as a constraint to adjust the risk thresholds within each category of individuals in order to indicate BP-lowering drugs to the mild hypertensives with the highest risk compared to their peers.

We examined the benefits and efficiency of our PB strategy against the 2007 ESH/ESC guidelines when implemented on the French primary prevention RVP, a one million individual-data base that reproduces the demographic composition of the nation in the age range 35–64 years and preserves the original co-variations of the individual characteristics, mainly modifiable and non modifiable cardiovascular risk factors[15]. We used the SCORE equations for low risk regions[16] to compute the 10-year fatal CVD risk for each individual (S1 Data, S2 Data). Virtual individuals were separated by sex and stratified by 10-year age categories. Primary outcomes were life-years gained in the years of potential life lost (relative life-year gain), and the number of subjects needed to treat to gain a life-year (NNT) by categories of gender and age. Simulations and all statistical analyses were performed in the R 2.14.1 statistical computing environment[17].

### Benefits, costs and efficiency indices

**Fig 1:**
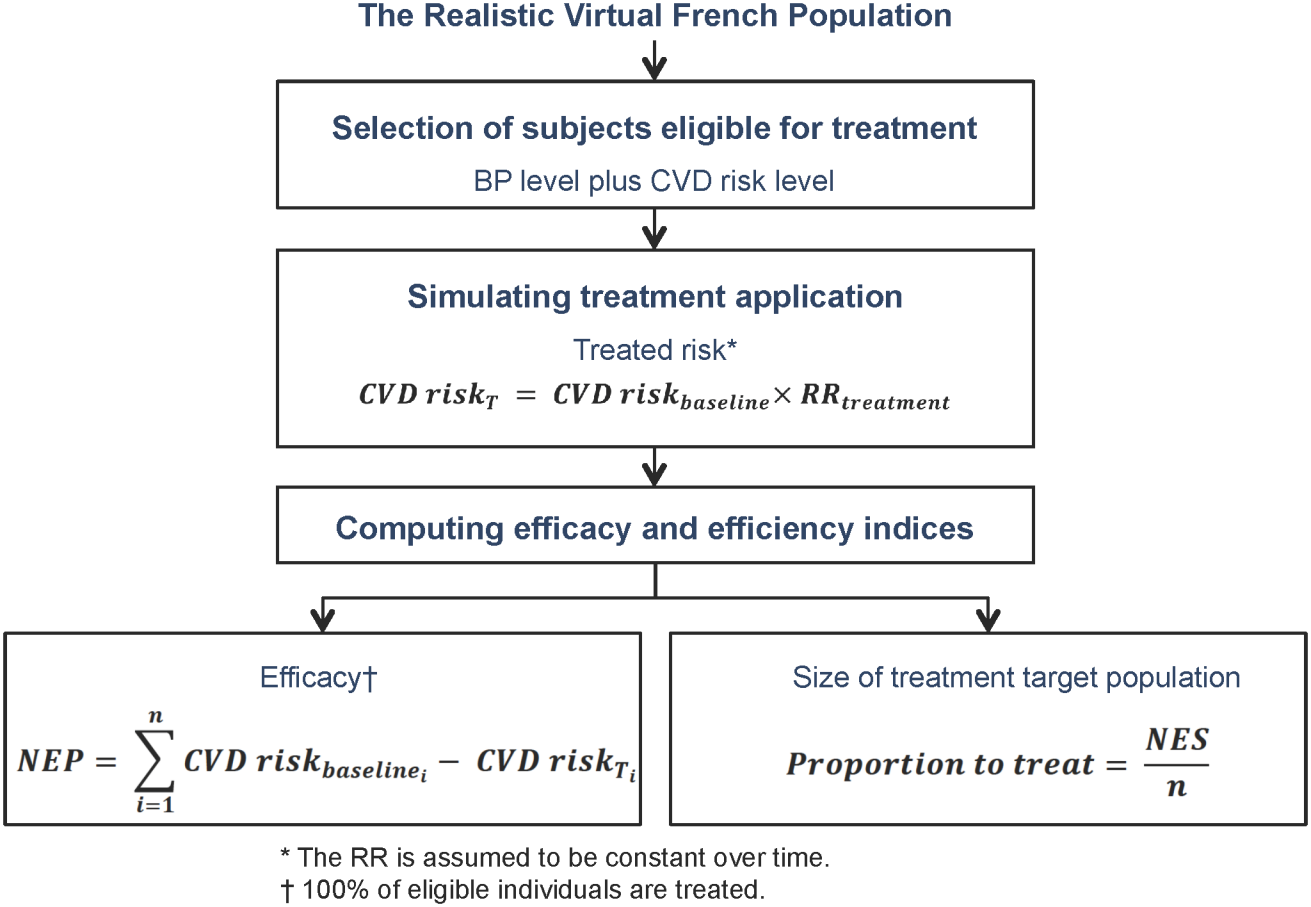
Procedure used to determine the proportions eligible and the number of events prevented thanks to treatment. Abbreviations: CVD, cardiovascular death; NEP, number of events prevented; NES, number of eligible subjects (Modified from reference 2).

In a previous work, we programmed a computing algorithm that: 1) selects population individuals having a BP level and CVD risk over a fixed threshold, 2) counts these individuals (number of eligible subjects, NES), 3) simulates their exposure to BP-lowering drugs and 4) estimates the number of events prevented (NEP) among them[2]. We assumed that the effect model that describes the relationship between the risk of CVD death without any treatment and the same risk modified by BP-lowering drugs[18] is linear. For each individual on BP-lowering drugs, the risk on treatment was obtained by multiplying its baseline risk by an average 0.83 relative risk of fatal CVD for both men and women derived from a meta-analysis of BP-lowering drugs trials[19], assuming no secular trend of effects (Fig 1). Health benefits were measured as the life-years gained[20], computed as the product of the number of events prevented by the treatment[2], and the 10-year life expectancy (Eq 1).

$$LYG_{age;sex}=NEP_{age;sex}\times \left(LE_{age;sex}-10\right)$$ (1)

Eq 1 allows computing the life-years gained over 10 years of treatment exposure. The benefit from treatment is the number of events avoided multiplied by the 10-year life expectancy. NEP and LE are, respectively, the number of events prevented and the mean life expectancy in the category.

We used the number of eligible subjects over a 10-year period as a proxy for costs and computed the efficiency as the number of subjects needed to treat over ten years to gain a life-year.

### Designing a new treatment strategy

We estimated the years of potential life lost—YPLL[21] from the number of cardiovascular deaths that would occur if no treatments were administered among the individuals potentially eligible and the 10-year mean life expectancy (Eq 2).

$$YPLL_{age;sex}=\Sigma_{i=1}^{n}CVDrisk_{i}\times \left(LE_{age;sex}-10\right)$$ (2)

Eq 2 to calculate years of potential life lost due to premature death of cardiovascular origin. CVD: fatal cardiovascular disease; LE: mean life expectancy estimated from French official statistics reported in 2010[22]; i: the individuals in the category.

We defined the proportional benefit as the ratio of the potential gain in life years under treatment over the expected years of life lost due to premature death of cardiovascular origin (Eq 3).

$$PB=\frac{LYG}{YPLL}$$ (3)

This proportional benefit estimated at the population level served as the reference for the equitable strategy. We computed the proportional benefit coefficient from the 2007 ESH/ESC guidelines implementation in the individuals potentially eligible of all ages and both genders, using the Eq 4:

PBESH/ESC=Σage=1;sex=13;2LYGage;sexΣage=1;sex=13;2YPLLage;sex (4)

### Computing proportional benefits for each category of individuals

In order to allocate the same proportional benefit to individuals of all categories of age and gender, we multiplied the burden of years of potential life lost class-specific by the PB_ESH/ESC_ coefficient to estimate the desired life-years gained (Eq 5, derived from Eq 3) and the number of events to prevent (NEP_des_) under the PB strategy (Eq 6, derived from Eq 1). Thus, among the youngest potentially eligible male about 400 deaths of cardiovascular origin were expected to occur in the next ten years, while 1600 deaths were expected in the second age category and 4000 deaths in the third category. Applying a PB coefficient of 10% implies that the desired number of deaths prevented would be, respectively, 40, 160 and 400.

$$LYGdes_{age;sex}=YPLL_{age;sex}\times PB_{ESH/ESC}$$ (5)

Eq 5 to compute the desired life-years gained by categories of gender and age according to the Proportional Benefit approach. It corresponds to the years of potential life lost multiplied by the overall ESH/ESC proportional benefit coefficient.

NEPdesage;sex=LYGdesage;sex(LEage;sex−10) (6)

Eq 6 used to calculate the desired numbers of events prevented over 10 years by age and gender categories, where NEP: number of events prevented; LYG: life-years gained; LE: mean life expectancy.

### Identification of risk thresholds adjusted by gender and ages

**Fig 2:**
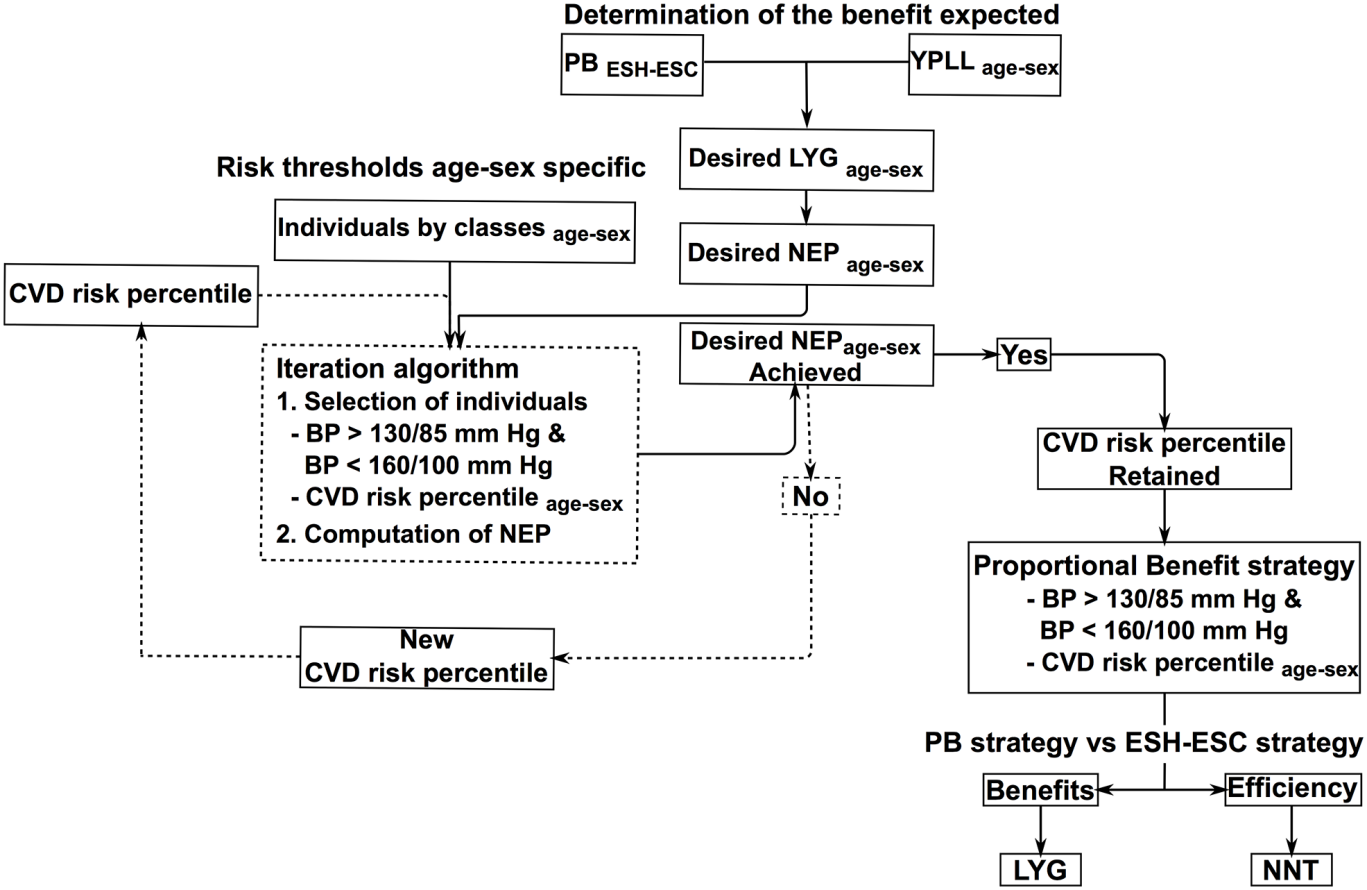
Simulation steps to design the proportional benefit strategy to identify the treatment target population. The reference is the overall Proportional Benefit, i.e. the relative life-year gain obtained from the application of the 2007 ESH/ESC recommendation on the potentially eligible individuals from the Realistic Virtual French Population. Abbreviations: PB, proportional benefit; YPLL, years of potential life lost; LYG, life-years gained; NEP, number of events prevented; NNT, number needed to treat to gain a life-year.

The function that simulates the exposure to treatment (Fig 1) was used to find the risk percentile that allows achieving the benefit desired NEP_des_ within each category of individuals potentially eligible. We tested by iteration an increasing risk percentile into the function until the *NEP obtained* was equal to the *NEP desired* (Fig 2). The resulting Proportional Benefit strategy thus recognizes the individuals to treat from the new CVD risk thresholds adjusted by ages and genders. In our example, these thresholds corresponded to percentiles 65, 63 and 61 in the first, second and third age category of virtual male.

### Comparing both treatment strategies and benefit ratios related to gender

We compared the PB strategy to the European recommendation of 2007 in terms of numbers of life-years gained and numbers of subjects needed to treat to gain a life-year upon ten years of treatment exposure. To account for the opportunity cost of investing at present rather than in the future considering that people give greater value to current health benefits over future benefits[13], the computations were done with and without discounting costs and benefits. A time preference rate of 3% per year was applied[14,23] to the 10-year life expectancy potentially recovered according to Eq 7. Future costs were discounted at the same yearly rate as health benefits accordingly with current NICE guidelines[12], assuming a constant cost-effectiveness threshold over time[9].

$$LE_{r}=\frac{1}{r}\times \left[1-\frac{1}{{\left(1+r\right)}^{\left(LE-10\right)}}\right]$$ (7)

Eq 7 To compute time preference rate applied to the life expectancy after 10 years of treatment exposure. With r: time preference rate and LE: life expectancy.

As a measure of how much the PB strategy reduces the differences of benefit between genders obtained applying the ESH/ESC recommendation, we computed the men/women ratios for the years of potential life lost without treatment and the benefits expected, expressed as NEP and LYG, at each 10-year age interval.

### Sensitivity analysis

The 2013 guidelines approach indicates that treatment is recommended if evidence is available and it may or should be considered when evidence is weak or unavailable. This is the case of grade 1 hypertension elderly patients and grade 1 hypertensives at low risk. We performed a sensitivity analysis to test to what extent the new recommendations could modify the proportions treated and benefits across the different categories of the potentially eligible population compared with the previous guideline version of 2007. We simulated the implementation of the new rule and assessed its impact on the NES and NEP, the number of residual events, i.e. the events that remained untreated in the individuals considered ineligible plus the events that remained despite BP lowering in treated individuals, the proportion of benefit and the efficiency. Elderly patients were identified as 60-year-olds and older individuals[24].

### Ethics Statement

Not applicable.

## Results

**Fig 3:**
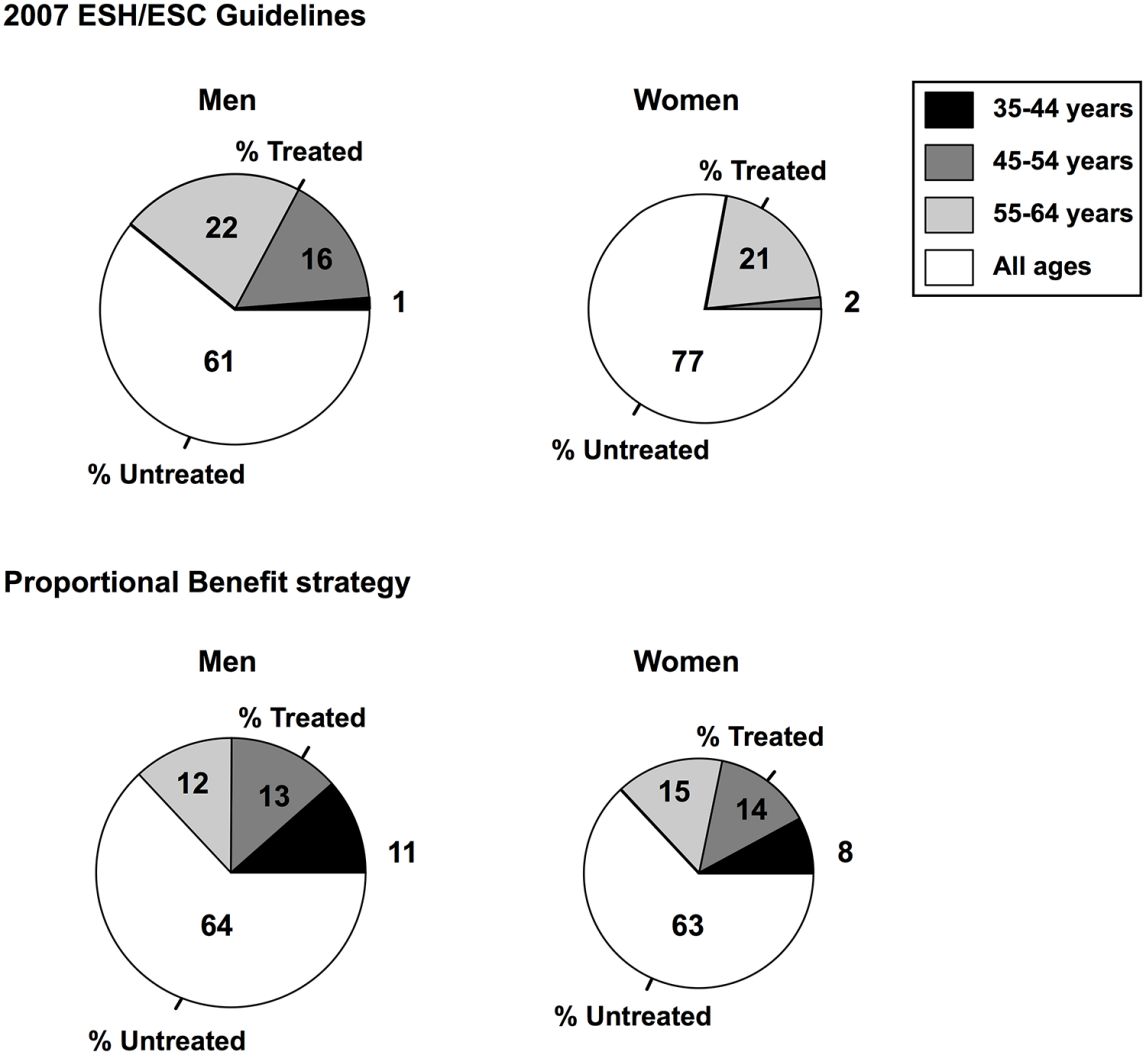
Age-related proportions of individuals to treat according to the PB strategy and the 2007 ESH/ESC guidelines. Pie areas indicate the relative contributions of age classes to the overall number of subjects eligible to treatment referred to the ineligible individuals of all ages.

The 2007 ESH/ESC guidelines provided 13074 life-years gained (NEP = 883) over 129199 years of potential life lost among the individuals potentially eligible (n = 466655), with a global proportional benefit coefficient of 10% (Table 2). The PB strategy resulted in a gender- and age-related gradient of risk-decision thresholds, with the highest threshold among individuals younger than 45 years and the lowest threshold in individuals over 55 years. While the proportions eligible to treatment showed a strong gradient related to age for both genders under the ESH/ESC strategy, this pattern was importantly attenuated in men and women by the PB strategy, with increased rates of treatment eligibility among individuals less than 55 (Fig 3). This implied an overall greater proportion of treated-to-untreated individuals under the PB strategy compared with the ESH/ESC strategy (37% versus 32% of the individuals potentially eligible on treatment, respectively).

**Table 2:** Sensitivity analysis assessing the impact of the 2013 ESH/ESC Guidelines on costs, proportion of benefits and efficiency.

		**Baseline**	**ESH/ESC 2007**		**PB strategy**		**ESH/ESC 2013**			
							Evidence-based		Opinion-based	
	**Age class**	**n**	**NES/n (%)**		**NES/n (%)**		**NES/n (%)**		**NES/n (%)**	
**Costs**	35–44	133.137	3		35		3		45	
	45–54	173.637	27		37		27		53	
	55–64	159.881	62		39		28		60	
	All	466.655	32		37		20		53	
		**NE**	**NEP/ NE (%)**	**NRE/ NE (%)**	**NEP/ NE (%)**	**NRE/ NE (%)**	**NEP/ NE (%)**	**NRE/ NE (%)**	**NEP/ NE (%)**	**NRE/ NE (%)**
**Benefits &**	35–44	435	2	98	10	90	2	98	9	91
**Residual**	45–54	1.990	8	92	10	90	8	92	10	90
**events**	55–64	5.421	13	87	10	90	4	96	11	89
	All	7.846	11	89	10	90	5	95	11	89
		**YPLL**	**LYG/YPLL (%)**		**LYG/YPLL (%)**		**LYG/YPLL (%)**		**LYG/YPLL (%)**	
**Proportion**	35–44	13.272	2		10		2		9	
**of benefit**	45–54	43.570	8		10		8		10	
	55–64	72.357	13		10		4		11	
	All	129.199	10		10		5		11	
			**NNT**		**NNT**		**NNT**		**NNT**	
**Efficiency**	35–44		144		347		146		515	
	45–54		131		144		134		207	
	55–64		107		84		144		117	
	All		114		131		139		179	

Abbreviations: n, number of individuals potentially eligible; NES, number of eligible subjects; NE, number of events among the individuals potentially eligible; NEP, number of events prevented by BP-lowering drugs; NRE, number of residual events; YPLL, years of potential life lost; LYG, life-years gained; NNT, number needed to treat to gain a life-year

**Fig 4:**
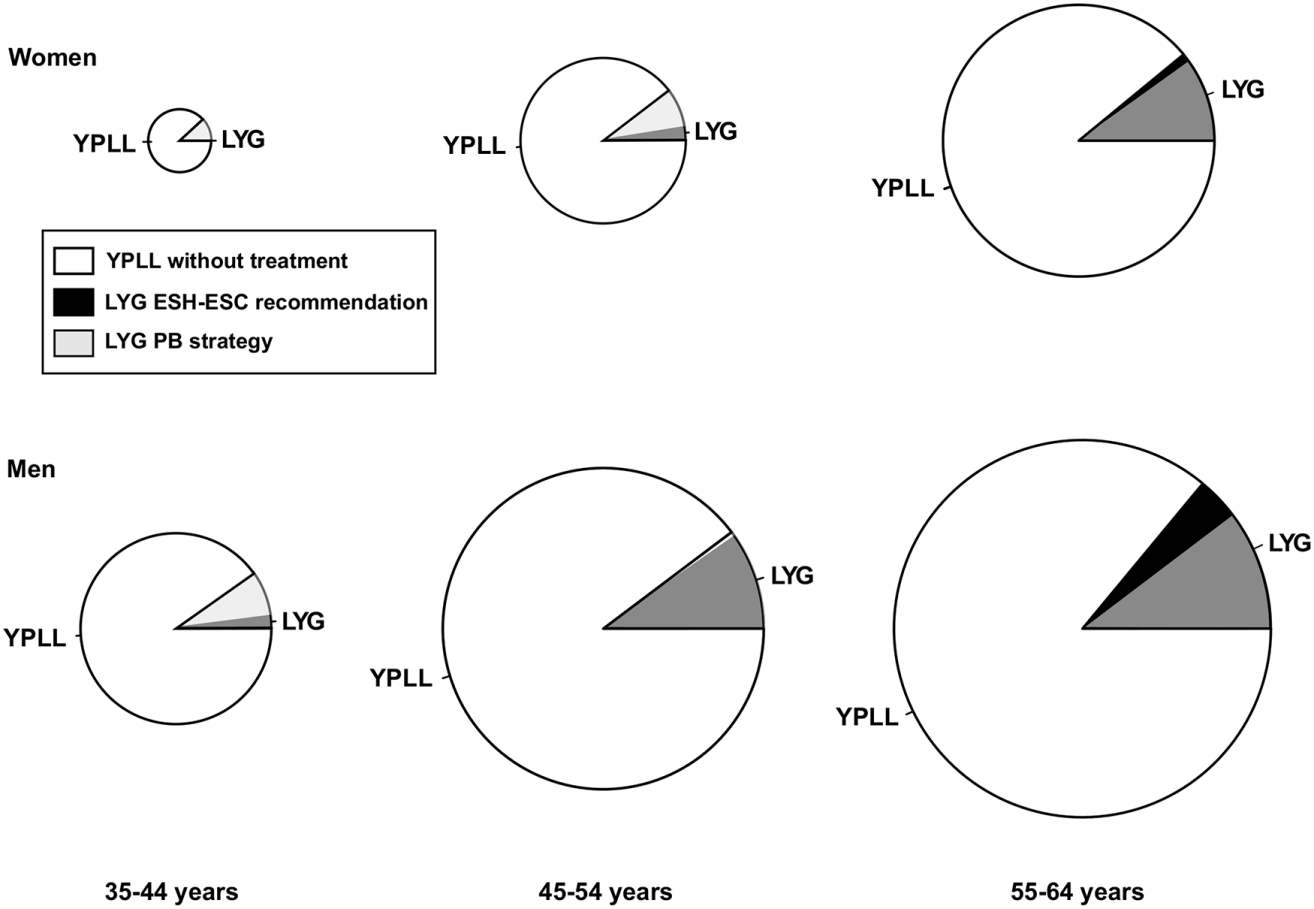
Rates of life-years gained referred to the rates of years of potential life lost by age classes obtained with the PB strategy and the 2007 ESH/ESC guidelines. Pie areas represent the LYG rates on treatment referred to rates of YPLL if no treatment were administered among the individuals potentially eligible within each sex and age category. Gains for either treatment strategy are shown together and partly overlapped in all categories except for women younger than 45 years. Pie radii scale indicates the age-related gradient of YPLL.

Fig 4 shows the life-years gained as well as the years of potential life lost among the individuals potentially eligible under the ESH/ESC guidelines and the PB strategy. While the ESH/ESC guidelines allocate most of gains to the oldest men (relative life-year gain = 14%) with no benefit for women less than 45 years, health gains are proportional to the potential losses in all categories under the PB strategy (relative life-year gain = 10%); this implied increased numbers of life-years to preserve and thus greater NEP_des_ at younger ages with slightly decreased numbers in men and women over 55 years of age. The PB strategy yielded virtually the same number of life-years gained, as did the European rule.

The sex-related benefit ratios decreased with the PB strategy compared to the ESH/ESC strategy in all age classes. As expected from the method we used, the men/women ratios of life-years gained with the PB strategy were equal to the naturally expected men/women ratios of years of potential life lost without treatment (Table 3). The PB strategy was more efficient (NNT = 131) than the 2013 ESH/ESC guidelines, whatever the level of evidence of the scenario adopted (NNT = 139 and NNT = 179 with the evidence-based scenario and the opinion-based scenario, respectively), although the 2007 ESH/ESC guidelines remained the most efficient strategy (NNT = 114). Discounting costs at the same rate as benefits decreased the efficiency for either treatment strategy. The PB strategy was more sensitive to this assumption than the ESH/ESC recommendation (relative change in NNT: -13% versus -9%).

**Table 3:** Gender-related ratios of potential life lost and gained according to the treatment strategy used.

Men/ women	Treatment strategy	Age classes (years)			
ratio		35–44	45–54	55–64	All
**YPLL**	Without treatment	9	3,7	1,9	2,6
**LYG**	ESH/ESC guidelines ^a^	∞	16	2,4	3,5
	PB strategy	8,3	3,7	1,9	2,6
**NEP**	ESH/ESC guidelines ^a^	∞	19,8	3,4	4,3
	PB strategy	10	4,6	2,7	3,2

^a^ Men/women benefit ratios with the 2007 European guidelines tend to infinity at ages 35 to 44 years since among women gains are equal to zero before the age 45 years.

Abbreviations: YPLL, years of potential life lost; LYG, life-years gained; NEP, number of events prevented.

### Sensitivity analysis

Implemented on the same population of potentially eligible individuals, the scenario with the lowest level of evidence according to the 2013 ESH/ESC guidelines, i.e., the opinion-based scenario, dramatically amplified the NES among the lowest risk individuals with 1472-fold more women to treat under the age 45 years, whereas the evidence-based scenario reduced to less than a half the NES among the oldest men and women compared to the 2007 guidelines (results not shown). In individuals of both sexes younger than 55 the NES was amplified by three under the opinion-based scenario, and virtually maintained by the evidence-based scenario, compared to the 2007 ESH/ESC guidelines. The LYG under the opinion-based scenario was 1,5-fold the LYG expected under the 2007 guidelines before the age 55 years; whereas in individuals aged 55 years and older the evidence-based scenario reduced this gain to one-third the LYG expected under the 2007 guidelines. The proportion of benefit, i.e. the relative life-year gain, was augmented in individuals younger than 55 years in the opinion-based scenario and reduced by more than two thirds at ages over 55 years in the evidence-based scenario, compared to the 2007 guideline version. The overall proportion of benefit from the 2007 guidelines was halved by the 2013 guidelines evidence-based scenario and increased by the opinion-based scenario (Table 2). The residual number of events was globally increased with the evidence-based scenario and decreased with the opinion-based scenario compared with the 2007 guidelines. The number needed to treat to gain a life-year over 10 years of drug treatment was greater under the 2013 guidelines disregarding the level of evidence of the scenario adopted compared to either the 2007 guidelines or the PB strategy (Table 2).

## Discussion

We propose a strategy whose benefit is proportional to the specific events incidence within each category of gender and age of a population, addressing the issue of the inequity induced by the high-risk approach with a number of practical consequences. The PB strategy reduced the sex-related benefit ratios at all ages, reflecting the actual differences on CVD incidence rates between men and women. The age-related gradient of benefit was abolished under the PB strategy. Identifying the treatment target population from the incidence rates of cardiovascular death, which are easily available from vital statistics in most of countries, appears more natural and fair than the gender or generation preferences induced by the application of absolute risk thresholds arbitrarily determined. The sensitivity analysis illustrates how simulation allows measuring the impact of recommendations. We have shown how the 2013 ESH/ESC evidence-based scenario reduces treatment coverage and benefits in elderly patients in the mild hypertensive range, in contrast to decisions based on the opinion of experts, which maintain rates of residual events at levels that are similar to the 2007 guideline version.

### Strengths and limitations of the study

Our strategy considers specific risk thresholds for age-sex categories as the unique additional rule besides BP levels to decide for treatment, instead of the various categories of individuals to treat proposed by the 2007 ESH/ESC guidelines. Simplified rules have the potential to improve physicians’ compliance to recommendations reducing the important gaps between recommendations for CVD management and actual clinical practice reported by observational studies[25].

As the benefit depends on the burden of disease, there is no reason to fix a unique budget for preventing CVD in every population and a re-calibration of risk estimation systems is promoted by the ESH/ESC guidelines to allow for secular trends in CVD mortality in different countries. Our approach allows adjusting the eligibility to treatment to the specific population background in terms of demography, the burden of disease and resources instead of calibrating the risk score in support of a suitable application of the absolute risk rule.

The PB strategy results in lower therapeutic coverage of people at high risk compared to the 2007 ESH/ESC strategy; of note, under this strategy, 67% of treated individuals of both genders were older than 55. However, according to the latest ESH/ESC guidelines, in elderly patients drug treatment should be initiated when SBP is greater or equal to 160 mm Hg. This implied that at the age of 60 years and over no one would have an indication of drugs among the mild hypertensive individuals included in our analysis and the proportion of 55-year-olds and older among the individuals treated decreased to only 24%. The PB strategy occupied a position in-between.

Regarding equity, concerns have been made on whether resources should be concentrated on the patients who can benefit most[26]. In a modelling study that estimated the benefits and costs of different prevention activities to reduce the burden of CVD in the US, lowering LDL cholesterol in people with existing coronary artery disease offered the largest benefit per person in terms of absolute myocardial infarction risk reduction. However, for the population as a whole this benefit was relatively small compared to other preventive activities given the reduced number of individuals who were candidates to this measure[27].

### Main consequences of the equitable proportional benefit strategy

The allocation of a constant relative benefit whatever the age or sex of the individuals is in line with the evidence regarding the effect of BP lowering drugs. Two recent meta-analyses of BP lowering trials stratified by levels of cardiovascular risk [28,29] indicate that the relative reduction of cardiovascular events obtained by BP lowering does not change at different baseline levels of cardiovascular risk and therefore, the absolute benefit increases with increasing risk. The PB strategy maximises gains per categories by allocating drug treatment to the individuals at the highest risk relative to their peers.

The PB strategy increases the therapeutic coverage of people with relatively low risk, i.e. young and women, who have also the greatest life expectancy. As an obvious consequence, the gain in life-years was increased in this subgroup, while the overall number of deaths prevented was reduced compared with the 2007 ESH/ESC guidelines that concentrate prevention efforts on the oldest men (Table 2). Considering the expected age-dependent decrease in QALYs[30] given the lifespan reduction and assuming that utility values per health state decrease with age and co-morbidities, redistributing the number of deaths prevented under the PB strategy would result in greater gains of quality-adjusted life years compared to the ESH/ESC recommendation. Opposite to the results of our equitable treatment rule, Liew and colleagues[14] have suggested that for individuals with different life expectancies but the same short-term CVD risk, treatment makes little difference on health benefits due to the counterbalancing effects of time preferences, case fatality rates and competing risks. It is thus necessary to provide a definition of what benefit is. From a societal perspective, discounting life expectancies by time preference rates implies giving relatively higher value to elders’ lives compared to the remaining lives of younger individuals, as indicated by the reported 40% reduction in life expectancies after discounting at ages 35–39 (from 41.7 to 23.6 years) compared to an 8% reduction at 85 years and over (from 4.87 to 4.47 years). But does the naturally higher vulnerability to death of the individuals that have already lived longer really make worthier treating them than treating younger individuals?

Based on the 2007 ESH/ESC recommendations, the PB strategy considers initiation of antihypertensive treatment when BP is in the high normal range and CVD risk is high compared to the peers’ risk. Drug treatment of high normal BP individuals has proven to be efficacious to reduce the incidence of hypertension with promising substantial public health effect, but the evidence in favour of early interventions is still considered limited. The recommended lifestyle measures for BP control in prehypertension have no demonstrable effect on public health. Assessing how far the benefit of early drug intervention lasts is key to address the issue of treatment eligibility in high normal BP subjects.

Modelling studies have shown that treating hypertension is cost-effective in different populations[31,32], with greater efficiency at increased age of treatment onset[32]. This is not surprising since age increases cardiovascular risk and thus maximises the gains per cost unit. The efficiency, as defined in the present study, was reduced with the PB strategy compared with the 2007 European recommendation, because a greater number of individuals have to be prescribed BP-lowering drugs to extend the benefits to the youngest and especially women. Nevertheless, it has also been shown that for persons treated with hypertension interventions, life extension could be achieved without increasing average lifetime medical spending[31]. Moreover, reducing morbidity as a result of hypertension treatment would still reduce health spending due to the avoided costs of informal care after an event[32].

The PB strategy offers an intermediate point of view between the classical high-risk approach promoted by historical guidelines and the latest hypertension guidelines that introduce new evidence-based recommendations. While the classical approach is the most efficient, the forecasted effectiveness and efficiency of the newest guidelines appear to be hardly competitive against this widely accepted reality. Our original approach has the merit of redistributing the same overall theoretical benefit of the high-risk approach within a population providing the same proportional benefit to every category of individuals, while improving the profile of residual events in younger individuals without elevating costs dramatically. The redistribution of benefit that reduces the residual risk at younger ages is in line with the conclusions of Thomopoulos et al., which promote extending treatment to low-to-moderate risk hypertensive patients in order to prevent the failures due to delayed onset of treatments[28].

### Implications for clinicians and policymakers

The current approach combines the absolute risk rule captured by the overall benefit expected from the application of the 2007 ESH/ESC guidelines used as a constraint to design the PB strategy with the application of the peers’ risk previously suggested. Other indices could also serve as constraints, such as the resources allocated to cardiovascular prevention at the scale of a country population, thus determining the maximum allowed number of eligible subjects to be distributed across the pertinent categories of individuals according to the proportional benefit definition.

The PB strategy is based on the discrimination ability of the risk equations used and, similarly to the risk age approach[1], calibration is indeed irrelevant to the rule application. Discrimination has been shown to be consistent across populations despite their different absolute risks[33–40] extending interestingly the potential applications of our methodology to other populations, disregarding the risk estimation system used as long as it has reasonable discriminatory power. Adapting the treatment rule needs to identify the CVD risk thresholds class-specific that lead to save a prefixed proportion of life-years in the years of potential life lost across the categories of individuals; even if the risk equation overestimates or underestimates CVD risk, this proportion would be unaffected. The CVD risk of the individual patient could be referred to the treatment rule established for that risk predictor in the whole country population until suitable coefficients are identified to calibrate the prediction.

### Perspectives

Our approach has the merit to highlight several dimensions in the decision process, which can be considered as independent: 1) effectiveness, 2) economics, which is combined with the first dimension into efficiency aspects; and 3) level of evidence: this dimension is illustrated by the fact that when guidelines promoted treating people with BP between 140/90 mm Hg and 160/95 mm Hg, any good level of evidence was lacking[41]. Our modelling approach should take this dimension into account in further developments. In next steps, we envisage demonstrating the generalizability of our approach by adapting the current recommendation to identify the individuals eligible to treatment in the Chilean population.

## Conclusion

We propose a new strategy to identify the individuals eligible to treatment defined in terms of the proportional benefit desired (or allowed) both at the scale of a country population and within each category of gender and age of that population. This method allows adapting recommendations to the risk factors profile and the incidence of cardiovascular events of a particular country or region. The PB strategy deals with the risk differences between genders and across ages taking into account the relative weight of CVD events according to the demographic structure instead of the overall population events only.

The Proportional Benefit strategy appears to be less efficient than the 2007 ESH/ESC guidelines. However, it offers an intermediate position with regards to the efficiency expected with the application of the latest ESH/ESC guideline version. Opposite to the treatment preference towards oldest men resulting from historical European recommendations, the PB strategy distributes fairly the benefit expected from treatment to all individuals from a population, regardless their age or gender. These contradictory pictures add new evidence to be balanced in decision-making: which price are we willing to pay for equity?

## Supporting Information

S1 Data The French Realistic Virtual Population database. It contains the data of every virtual individual (rows) characterised by 16 covariates (columns).
(ZIP)

S2 Data A description of the covariates included in S1 Data. (TXT)
